# Supplementary material for: Meiotic DNA exchanges in C. elegans are promoted by proximity to the synaptonemal complex
Source: Life Sci Alliance. 2023 Jan 25;6(4):e202301906. doi: 10.26508/lsa.202301906 (PMC9877436; doi:10.26508/lsa.202301906)
Supplement: Supplementary file 1 [file LSA-2023-01906_TableS1.pdf]

Table S1: Summary of sister exchanges and GFP-COSA-1 foci counts

	sister exchanges, paired chromosomes				sister exchanges, unpaired chromosomes							
Genotype	Total	Non-Exchanges	One exchange	% exchange chromatids	Total	Non-exchanges	One exchange	Two exchanges	% exchange chromatids	Sister exchanges per nucleus[b]	GFP-COSA-1 foci per nucleus	Source
wildtype	49	47	2	4.1%	-	-	-	-	-	0.5	5.9	This work and (Almanzar et al., 2021)
him-8	35	35	0	0%	24	23	1	-	4.2%	0.1	5.2	This work and (Almanzar et al., 2021)
zim-2	34	30	4	11.8%	10	9	1	-	10%	1.4	-	This work
ieDf2	-	-	-	-	182	114	65	3	39.0%	4.7	4.8	This work
rec-8	-	-	-	-	7	3	4		57.1%	6.9	10.4	(Almanzar et al., 2021; Cahoon et al., 2019)
syp-3 (me42)	-	-	-	-	33	15	14	4	66.7%	8.0	7.4	(Almanzar et al., 2021)
syp-1^{K42E}	-	-	-	-	64[a]	24	29	11	79.7%	9.6	16.4	This work and (Almanzar et al., 2021)
syp-1^{K42E} *zhp3(-)*	-	-	-	-	67	46	21	-	31.3%	3.8	-	This work
syp-1	-	-	-	-	55	34	21	-	38.2%	4.6	3.5	This work
syp-2	-	-	-	-	30	20	9	1	36.7%	4.4	4.6	This work and (Cahoon et al., 2019)
nT1/+	23	21	2	8.7%	-	-	-	-	-	1.0	6.0	This work
hT2/+	14	14	-	0%	-	-	-	-	-	0.0	-	This work

[a] Data is combined for *syp-1*^{K42E} worms and *syp-1*^{K42E} *ZHP-3-FLAG-AID* worms grown without auxin.
[b] Sister exchanges per nucleus are extrapolated from the exchange rates on paired and unpaired chromosomes as explained in the Methods.
